# The influence of varus and valgus deviation on the contact area of patellofemoral joint in healthy knees

**DOI:** 10.1186/s12891-023-06976-9

**Published:** 2023-10-31

**Authors:** Xiaomeng Wang, Lisha Duan, Huixin Liu, Hailang Ge, Zhenyue Dong, Xiaobo Chen, Chenyue Xu, Gang Ji, Huijun Kang, Fei Wang

**Affiliations:** 1 Foot and Ankle surgery, The Third Hospital of Hebei Medical University, Hebei, China; 2 Imaging Department, The Third Hospital of Hebei Medical University, Hebei, China; 3 Ultrasound Medicine Department, The Third Hospital of Hebei Medical University, Hebei, China; 4 Orthopedics Department, The Third Hospital of Hebei Medical University, Hebei, China; 5 Department of Joint Surgery, The Third Hospital of Hebei Medical University, Hebei, China

**Keywords:** Varus-valgus alignment, Patellofemoral joint, Contact area, Knee flexion

## Abstract

**Object:**

Varus-valgus lower alignment is a risk factor for patellofemoral osteoarthritis, but malalignment alone affect not only the tibiofemoral joint but also the patellofemoral joint. The aim of the present study was to analyse the contact area of patellofemoral joint in varus alignment and valgus alignment of healthy subjects using magnetic resonance imaging.

**Methods:**

Twenty-six healthy subjects with valgus lower limb alignment (Group I, n = 26) and twenty-six volunteers with varus lower limb alignment (Group II, n = 26) was performed. An MRI scan was used to capture and measure the patellofemoral joint articular cartilage contact area at different degrees of knee flexion (20°, 40°,60°) in passive movement. All subjects were categorized on the basis of the global limb alignment and mechanical alignment of the femur and tibia. Varus alignment is hip–knee–ankle angle ≥ 3°; and valgus alignment is hip–knee–ankle angle ≥ − 3°. To obtain medial facet contact area and lateral facet contact area for each slice, the length of each respective line of contact was multiplied by the 5 mm slice thickness.

**Results:**

The overall joint contact area increased from 168.0 ± 20.5 mm^2^ at 20° knee flexion to 334.4 ± 30.5 mm^2^ at 60° knee flexion in group (I) The overall joint contact area increased from 178.0 ± 18.9 mm^2^ at 20° knee flexion to 328.9 ± 27.2 mm^2^ at 60° knee flexion in group (II) There was a significant difference in lateral facet contact area between group I and group II at 40° of knee flexion. There was significantly different in medial facet contact area between group I and group II at 20° and 40° of knee flexion.

**Conclusions:**

Throughout the knee movement, the contact area on the lateral facet of the patellofemoral joint was greater in the valgus group. In the early phase of knee flexion, the contact area of the medial patellofemoral joint was larger in the varus group. Lower alignment is an important factor in patellofemoral joint degeneration.

## Introduction

The patellofemoral joint is geometrically complex, asymmetric and strongly influenced by kinematic forces around the knee. Understanding the changes in the kinematics of the patellofemoral joint can help explain both the cause and treatment of patellofemoral joint disorders [1]. We believe that the patellofemoral joint contact area and pressure have more influence on the cartilage of the patellofemoral joint. The determination of the location and size of the joint contact area is the congruency of the patellofemoral joint [2].

There are many factors that influence the kinematics of patellofemoral joint, including bony structures, soft tissue anatomy, and limb alignment. Lower alignment is a key determinant of load distribution, and both varus and valgus alignment contribute to the progression of osteoarthritis (OA) [3, 4]. In a previous study, Cahue described that valgus alignment leads to a higher progression of patellar osteoarthritis than varus alignment, especially on the lateral part of the patella [5]. A recent study has shown that valgus malalignment is associated with progression of lateral patellofemoral osteoarthritis, and varus malalignment with osteoarthritis of the medial patellofemoral joint [5, 6]. It is believed that the alignment is related to the degeneration of the joint and the area of cartilage wear. Many scholars have studied tracking differences with varus and valgus in arthritis patients [7–9]. But this is already a biomechanical measure after degeneration, so is there a difference in normal healthy knees? There are few studies of the patellofemoral joint contact area in lower valgus and varus alignment in healthy people.

Magnetic resonance imaging (MRI) and three-dimensional finite element have been shown to be valid methods for quantifying patellofemoral joint contact area, indicating the potential for in vivo assessment [10, 11]. MRI has the advantages of simplicity and ease of implementation compared to three-dimensional finite element. The purpose of the present study was to analyse the patellofemoral contact area for varus and valgus of healthy knees using magnetic resonance imaging.

## Patients and methods

### Participants

All participants gave written informed consent and approval for this study was obtained from the Ethics Committee of the Third Hospital of Hebei Medical University (2023-002-1). The participants were divided into two groups according to the lower alignment: varus group and valgus group.

Group size was calculated by a two-sample independent samples Student’s t-test with an effect size of 0.8, 80% power, type I error = 0.05, and a sampling ratio of 1:1 (case:control groups) resulting in a minimum required sample size of 26 patients in each group.

Inclusion criteria: (1) 18 years<age<35 years, patients with no history of lower extremity trauma, surgery or systemic disease, (2) Alignment of knee joint: 7°>varus > 3°, 7°>valgus > 3°, (3)18 kg/m^2^ < body mass index(BMI) < 34 kg/m^2^. Exclusion criteria: (1) patients with degenerative knee joint disease or patellofemoral dysplasia, (2) inability to perform MRI (internal fixation, claustrophobia), (3) symptoms such as knee pain, swelling, and/or dislocation, (4) suppurative, rheumatoid, or tuberculous arthritis, (5) intraarticular tumour or tumour-like lesion. A total of 6 subjects were excluded. Three patients were excluded because of knee cartilage injury, two patients were excluded because of knee symptoms, and one patient was excluded because of MRI could not be completed.

Twenty-six healthy individuals with valgus lower limb alignment (Group I, n = 26) and nineteen volunteers with varus lower limb alignment (Group II, n = 26) were studied. An MRI scan was used to capture and measure the patellofemoral articular cartilage contact area at different degrees of knee flexion (20°, 40°,60°) in passive movement.

Right knee motion was analysed in all subjects. Gender, age and BMI (Body Mass Index) were well matched between the group I (vaglus alignment) and group II (varus alignment). BMI was measured as weight (kg)/height (m^2^).

### Measurement of alignment

Standing full-limb radiographs of all eligible knees from participants were obtained at the Third Hospital of Hebei Medical University. These radiographs were read for mechanical axis knee alignment in all participants. The minimum malalignment for inclusion in this study was 2° in the varus or valgus direction from neutral alignment defined as 0°.

All subjects were categorized based on global limb alignment and mechanical alignment of the femur and tibia. Varus alignment is HKAA ≥ 3°; and valgus alignment is HKAA < − 3°. The angles measured: hip–knee–ankle angle (HKA, the angle is formed by the lines connecting the centres of the femoral head, the knee and the talus).

### Measurements of patellar size and shape of trochlear groove

Trochlear groove was determined by MRI cross-section, trochlear dysplasia by Dejour [12], and the patellar height was defined by the Caton-Deschamps-index [13]. Measurement of patellar height (Insall–Salvati ration): The ratio of the length of the patella to the length of the patellar tendon. Patellar height was measured from reconstructed sagittal plane images.

### Patient position

The patient was placed in the supine position with a fixed Angle adhesive pillow placed under the knee joint. Knee flexion was 20°, 40°, and 60°. Data were captured in passive movement with quadriceps contraction.

### MRI scanning protocol

This MR imaging method of assessing contact area has been shown to be reliable and comparable with contact area measurements obtained using Fuji pressure sensitive film in cadaver specimens [14].

MR imaging was performed on a 1.5 T Siemens MR scanning system (Altea, Siemens, Erlangen, Germany) with patients lying in a supine position and the large body matrix coil was used. Three-dimensional turbo fast low angled shot (FLASH) T1 weighted images (3D T1WI) was acquired with the following parameters: repetition time/echo time, 19.88/7.34 ms; flip angle, 25°; number of slices, 96; slice thickness/gap, 0.8/0.16 mm; acquisition matrix, 180 × 180; field of view, 154 mm×154 mm; scan time, 02:53 min.

To ensure that all patellofemoral contact images were captured, the entire trochlea and articular surface of the patella were included in the scan range. The demarcation point of the medial and lateral articular surfaces was the median patellar ridge. To obtain medial facet contact area (MFCA)and lateral facet contact area (LFCA)for each slice, the length of each respective line of contact was multiplied by the 5-mm slice thickness. The contact areas from each sequential image were summed to obtain the patellofemoral joint contact area for each facet. Total facet contact area (TFCA) was calculated by summing the medial and lateral facet contact areas at each knee flexion angle. Contact area measurements were made twice by the same investigator and averaged for final analysis.

### Image interpretation

**Fig. 1:**
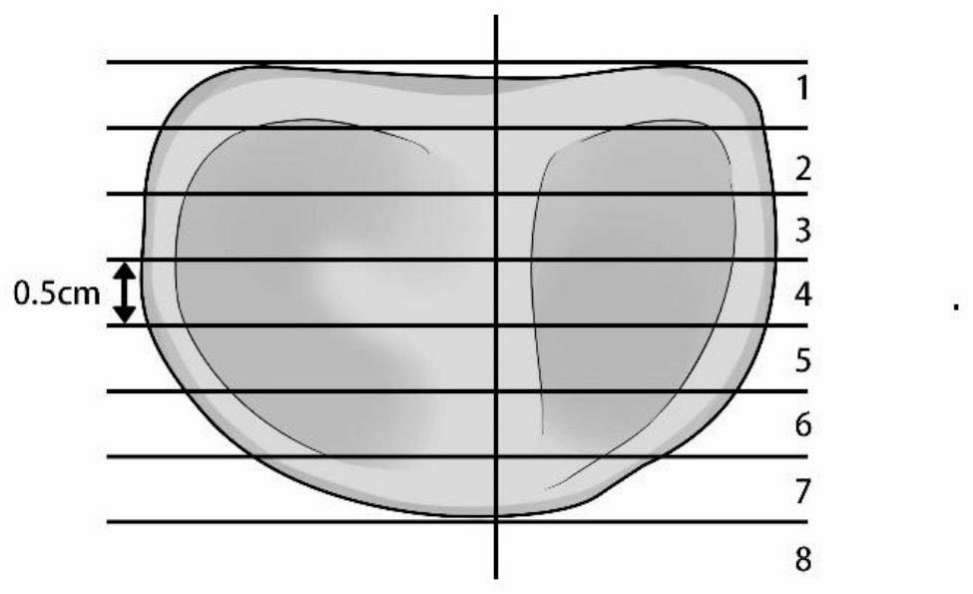
The diagram of area measurement ‘Flattening’ of axial imaging. Slices are at 5 mm intervals. The width of each slice in which there is contact between the femur and patella cartilage is multiplied by 5 mm

Using digital data from the institutional PACS (Donghua Healthcare Centricity, Beijing, China), the operator manually identified the boundaries of articular cartilage contact between the patella and femur on both sides of the trochlear sulcus in axial view [10]. These measurements were recorded as linear distances. MRI scans were performed 5 mm apart from each layer, and the cartilage contact area of each layer was calculated by multiplying the cartilage contact length by 5 mm. Each slice surface is then summed to reach a measure of the area in contact between the articulating surfaces to provide a measure of congruence in each measurement condition. This was recorded on a ‘flattened’ 2D coronal plane image for visual interpretation of the contact area [10] (Fig. 1).

### Statistics

All statistical analyses were performed using *SPSS* 26.0 (IBM Corp., Armonk, New York). Each of the measures were calculated by descriptive statistics. Quantitative variables were described by means and standard deviations (S ± D). A two-sample *t*-test and chi-squareor or Fisher test for continuous variables and categorical data were used to examine the differences in age, BMI and gender respectively. The significance level was set at *P* < 0.05. To determine whether contact area varied between different knee flexion angles, an analysis of variance (ANOVA) with repeated measures was performed for each dependent variable. The two-sample *t*-test was used to compare the differences in patellofemoral joint contact area between the varus and valgus groups.

## Results

The average ages were 24.1 ± 3.7 years for the group I and 25.0 ± 3.9 years for the group II. There was no statistical difference in ages between the genders (*p* = 0.473). The average BMI of the were 26.1 ± 4.2 for group I and 25.3 ± 4.0 for the group II. There was no dysplasia in the trochlear groove to Dejour in group I and group II. The average patellar height (Insall–Salvati ratio) were1.08 ± 0.17 for group I and 1.04 ± 0.13 for the group II. (Table 1)

**Table 1:** Characteristics of the group of varus alignment and the group of valgus alignment

	GroupI(n = 26)	Group II (n = 26)	t(χ2) (95% CI)	*P*
Age	23.4 ± 3.5	24.1 ± 3.8	-0.689	0.494
Gender(M\F)	15/11	14/12	0.78	0.78
BMI (kg/m^2^)	26.1 ± 4.2	25.3 ± 4.0	0.716	0.477
PH	1.08 ± 0.17	1.04 ± 0.13	0.834	0.403

Group I :the group of valgus alignment Group II : the group of varus alignment

BMI : Body Mass Index PH : patellar height

### Trends in overall joint contact area

The overall joint contact area increased from 168.0 ± 20.5 mm^2^ at 20° knee flexion to 280.8 ± 29.4 mm^2^ at 40° knee flexion and to the 334.4 ± 30.5 mm^2^ at 60° knee flexion in the group I. The overall joint contact area increased from 178.0 ± 18.9 mm^2^ at 20° knee flexion to 281.4 ± 23.3 mm^2^ at 40° knee flexion and to the 328.9 ± 27.2 mm^2^ at 60° knee flexion in the group II.

**Fig. 2:**
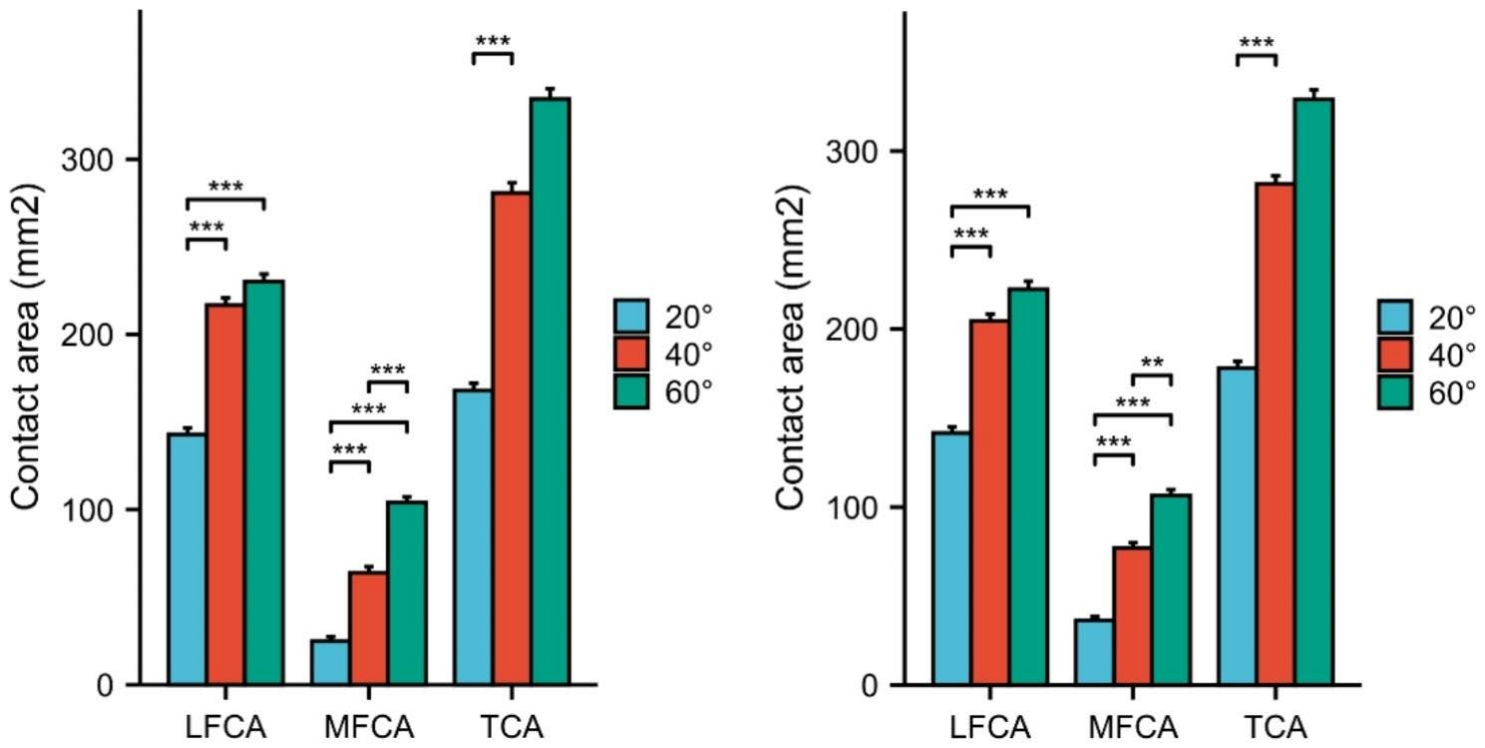
Graphs show the changes in contact area in group I and group II at different knee flexion LFCA: lateral facet contact area MFCA: medial facet contact area TFCA: total facet contact area Group I :the group of valgus alignment Group II : the group of varus alignment

In both group I and group II, all joint contact areas increased with the increase of knee flexion angle. There was a clear statistical difference between each knee flexion angle. (Table 2) (Fig. 2a and b).

**Table 2:** Comparison of mean joint congruence between different knee flexion angles

Contact area Angle of Knee flexion(°)	Group	LFCA(mm^2^)	MFCA(mm^2^)	TFCA(mm^2^)	*F*	*P*
20	Group I	143.0 ± 19.0	25.0 ± 13.5	168.0 ± 20.5	136.274	0.000
	Group II	141.6 ± 17.8	36.4 ± 10.9	178.0 ± 18.9	113.983	0.000
40	Group I	216.7 ± 20.7	64.0 ± 17.0	280.8 ± 29.4	166.597	0.000
	Group II	204.5 ± 19.6	77.0 ± 15.1	281.4 ± 23.3	152.975	0.000
60	Group I	230.2 ± 21.8	104.1 ± 16.2	334.4 ± 30.5	253.666	0.000
	Group II	222.3 ± 23.0	106.5 ± 16.9	328.9 ± 27.2	282.718	0.000

LFCA: lateral facet contact area MFCA: medial facet contact area TFCA: total facet contact area

Group I :the group of valgus alignment Group II : the group of varus alignment

### Medial facet contact area and lateral facet contact area

**Fig. 3:**
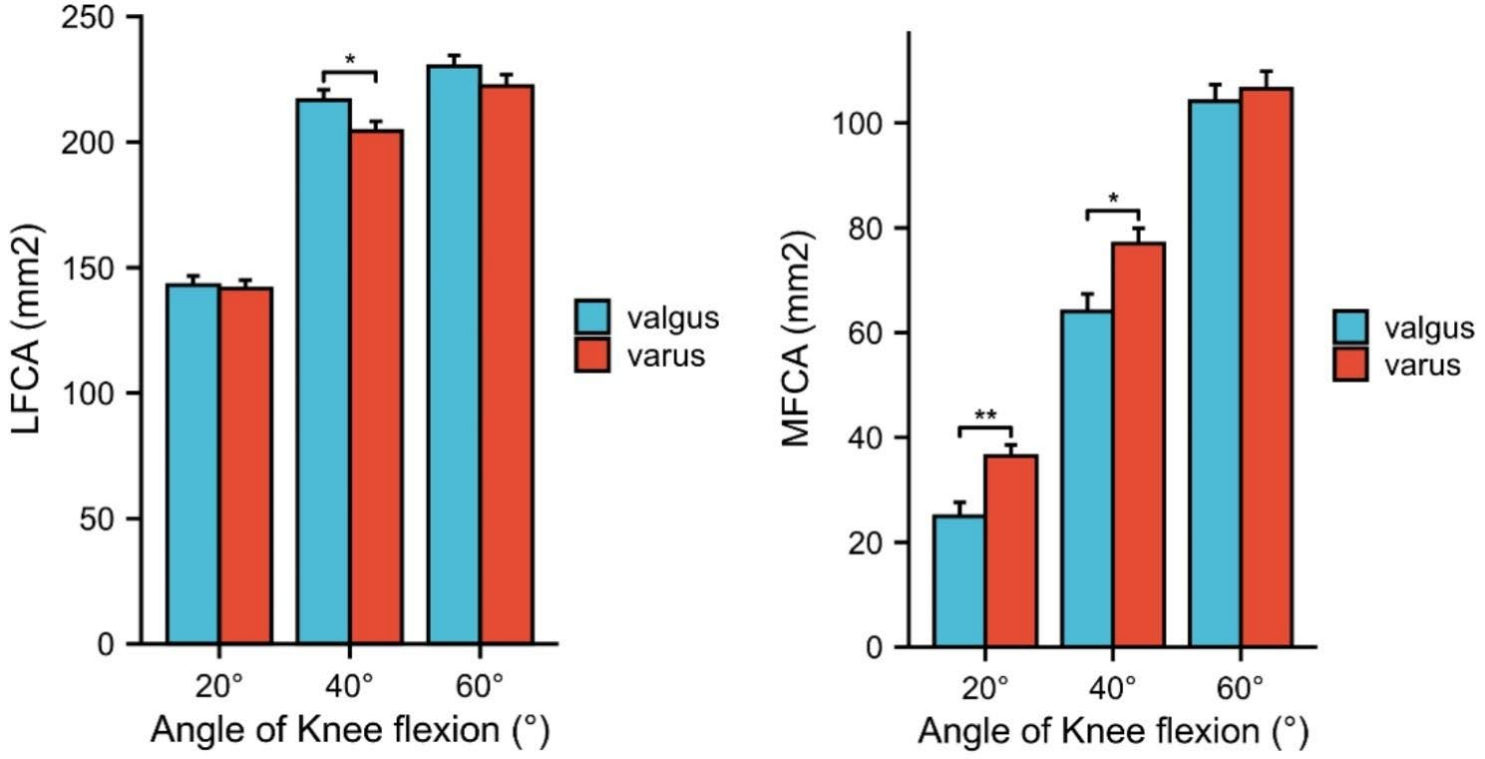
Histogram showing changes of contact area in different facets between group I and group II at different knee flexion LFCA: lateral facet contact area MFCA: medial facet contact area TFCA: total facet contact area Group I :the group of valgus alignment Group II : the group of varus alignment

The medial facet contact area increased from 25.0 ± 13.5 mm^2^ at 20° knee flexion to 104.1 ± 16.2 mm^2^ at 60° knee flexion in group (I) The medial facet contact area increased from 36.4 ± 10.9 mm^2^ at 20° knee flexion to 106.5 ± 16.9 mm^2^ at 60° knee flexion in group (II) The medial facet contact area was statistically different in each flexion angle in both group I and group II (20° ,40°,60°). (Fig. 3b)

The lateral facet contact area increased from 144.1 ± 20.2 mm^2^ at 20° knee flexion to 232.5 ± 22.8 mm^2^ at 60° knee flexion in group (I) The lateral facet contact area increased from 141.5 ± 19.9 mm^2^ at 20° knee flexion to 222.7 ± 25.0 mm^2^ at 60° knee flexion in group (II) The lateral facet contact area was statistically different in any flexion angle in group I and group II (20° ,40°,60°). (Fig. 3a)

There was no difference in total contact area between Group I and group II at all knee flexion angles. (Fig. 3c) There was significant difference in medial facet contact area between group I and group II at knee flexion 20° and 40°. (Fig. 3b) There was a significant difference in lateral facet contact area between group I and group II at 40° of knee flexion. (Table 3) (Fig. 3a).

**Table 3:** Comparison of mean joint congruence between the groups of varus and valgus

Contact area Angle of Knee flexion(°)	Group	LFCA(mm^2^)	*t*	*P*	MFCA(mm^2^)	*t*	*P*	TFCA(mm^2^)	*t*	*P*
20	Group I	143.0 ± 19.0	0.279	0.781	25.0 ± 13.5	-3.375	0.001	168.0 ± 20.5	-1.839	0.072
	Group II	141.6 ± 17.8			36.4 ± 10.9			178.0 ± 18.9		
40	Group I	216.7 ± 20.7	2.190	0.033	64.0 ± 17.0	-2.904	0.005	280.8 ± 29.4	-0.092	0.927
	Group II	204.5 ± 19.6			77.0 ± 15.1			281.4 ± 23.3		
60	Group I	230.2 ± 21.8	1.267	0.211	104.1 ± 16.2	-0.521	0.605	334.4 ± 30.5	0.683	0.498
	Group II	222.3 ± 23.0			106.5 ± 16.9			328.9 ± 27.2		

LFCA: lateral facet contact area MFCA: medial facet contact area TFCA: total facet contact area

Group I :the group of valgus alignment Group II : the group of varus alignment

## Discussion

The aim of this study was to determine whether contact area is associated with varus and valgus alignment in healthy subjects. With increasing of knee flexion angle, the patellofemoral contact area increased both in both the varus and valgus knee groups. In addition to this, we found that the varus group displayed greater medial facet contact area compared with the valgus group in early flexion of the knee (20° and 40°) and smaller lateral facet contact area compared with the valgus group in early flexion of the knee (40°).

The total patellofemoral joint contact area increased significantly with knee flexion angle increasing, which is consistent with the findings of previous investigations despite use of different methodologies [10, 15]. Using cadaver limbs and pressure sensitive film, Powers [16] reported a 68% increase in contact area between 15° and 60° of knee flexion, while D’Agata [17]reported an 81% increase between 20° and 60°. Between knee flexion 20° and 60°, an 80% increase in total contact area was observed in the Gretchen’s research [15]. In this study, approximately 99% of the total increase in contact area was achieved by 60° in group I and approximately 87%~99% of the total increase in contact area was achieved by 60° in group II.

In addition to similar percentage increases, the actual contact area values obtained in the current study were within the ranges reported in previous investigations. Our study adds to the evidence that the average total contact area of patellofemoral joint is 168.0 ~ 178.0 mm^2^ at 20° and 328.9 ~ 334.4 mm^2^ at 60°. Gretchen’s study confirms that the average total contact area of PFJ is 186 mm^2^ at 20° and 334 mm^2^ at 60° [15]. D’Agata et al [17] reported contact area values ranging from 160 mm^2^ at 20° to 290 mm^2^ at 60°, and Huberti and Hayes [3]reported measurements of 260 mm^2^ at 20° and 390 mm^2^ at 60°. The overall joint contact area increased from 168.0 ± 20.5 mm^2^ to 334.4 ± 30.5 mm^2^ in group I and 178.0 ± 18.9 mm^2^ to 328.9 ± 27.2 mm^2^ in group II with knee flexion (20°~60°) in this study. In addition, previous studies have shown that the greatest change in the increase of patellofemoral joint contact area is between 40° and 45° of knee flexion, which was confirmed in this study. In this study, approximately 58 ~ 67% of the total increase in contact area was achieved by 40°, whereas Powers et al. [16] reported a 100% increase in contact area by 45° knee flexion. Although the measurements were inconsistent, they all demonstrated that the greatest changes in contact area occurred between 20° and 40° of knee flexion.

Gross et al. [8]reported that knees with varus malalignment exhibited a higher prevalence of medial than lateral patellofemoral damage in all cohorts. Although patellofemoral osteoarthritis of the lateral facet of patellofemoral OA was induced in valgus knees, the mechanism behind the progression of patellofemoral OA is unclear, especially in varus knees [9]. A previous study found that varus knee deformity was associated with worsening of patellofemoral OA, and severe varus deformity mainly induced OA of the lateral facet [7]. McWalter concluded that varus and valgus malalignment do not mandatorily alter three-dimensional patellar kinematics [7]. Throughout the whole knee flexion process, the contact area of the lateral patellofemoral joint is much larger than that of the medial articular surface. This study confirms that the average contribution from the lateral facet was 68 ~ 84%, whereas only 16 ~ 32% came from the medial facet in both the valgus and varus groups between 0° and 60° knee flexion. According to the results, we believe that the higher incidence of patellofemoral OA is related to the larger contact area of the lateral facet of the patellofemoral joint during knee activities.

Knee alignment is a key determinant of load distribution. Our MRI scans demonstrated a difference in contact area between the varus and valgus groups. There was significant difference in contact area of medial facet between the varus and valgus groups at 20° and 40° of knee flexion. However, the contact area of lateral facet was not significantly different between the varus and valgus groups at 20° and 60° of knee flexion. In this study, this contact pattern can be explained by the anatomy and kinematics of the patellofemoral joint. As described by McWalter in a previous study, tibiofemoral malalignment would be expected to cause the patella in varus knees to track medially and the patella in valgus knees to track laterally [7].

The difference in the contact areas of the medial and lateral patellar articular surfaces between the varus and valgus groups was greatest when the knee was flexed at 40°. The differences in the medial and lateral articular surfaces between the varus and valgus groups were smaller at the other knee flexion angles. This phenomenon may be related to the characteristics of patella movement in varus and valgus knees. The patella in the varus group was tilted medially throughout the tested range of knee flexion, whereas the patella in the valgus group started with less medial tilt but tilted medially with knee flexion [18]. Another reason is that the patella enters the trochlear groove and the bony structure plays an important role.

There are some limitations. For ethical reasons and to avoid patient discomfort, three knee angles were selected for measurement in this study. The method used to calculate the contact area in this study, although validated, is not accurate enough. In this study, only a single plane was used to analyse the contact area, which has some shortcomings. Another limitation is the number of subjects in each group in this study. Despite the small sample size in this study, there were significant differences in the contact area between 20° and 40° of knee flexion, when comparing the varus and valgus groups.

## Conclusion

With increasing of knee flexion angle, the patellofemoral joint contact area increased in both the varus and valgus knee groups. At the early phase of knee flexion, the medial patellofemoral facet contact area was greater in the varus group. The difference in contact area was greatest between group I and group II at 40° knee flexion. This study confirmed that lower alignment affects the patellofemoral joint contact area and explains for the incidence of patellofemoral arthritis in varus and valgus alignment. Lower alignment is an important factor in patellofemoral joint degeneration.

## Data Availability

All data generated during this study are included in this published article and its supplementary information files.
